# Suboptimal patterns of provider initiated HIV testing and counselling, antiretroviral therapy eligibility assessment and referral in primary health clinic attendees in Blantyre, Malawi*

**DOI:** 10.1111/j.1365-3156.2011.02946.x

**Published:** 2012-02-01

**Authors:** Peter MacPherson, David G Lalloo, Augustine T Choko, Gillian H Mann, Stephen Bertel Squire, Daniel Mwale, Eddie Manda, Simon D Makombe, Nicola Desmond, Robert Heyderman, Elizabeth L Corbett

**Affiliations:** 1 Liverpool School of Tropical MedicineLiverpool, UK; 2 Malawi-Liverpool-Wellcome Trust Clinical Research ProgrammeBlantyre, Malawi; 3 Blantyre District Health OfficeBlantyre, Malawi; 4 HIV & AIDS Unit, Ministry of Health of MalawiLilongwe, Malawi; 5 London School of Hygiene and Tropical MedicineLondon, UK

**Keywords:** HIV, provider initiated testing and counselling, WHO clinical staging, antiretroviral therapy, programmatic evaluation, primary health care, qualitative

## Abstract

**Objective:**

To understand reasons for suboptimal and delayed uptake of antiretroviral therapy (ART) by describing the patterns of HIV testing and counselling (HTC) and outcomes of ART eligibility assessments in primary clinic attendees.

**Methods:**

All clinic attendances and episodes of HTC were recorded at two clinics in Blantyre. A cohort of newly diagnosed HIV-positive adults (>15 years) was recruited and exit interviews undertaken. Logistic regression models were constructed to investigate factors associated with referral to start ART. Qualitative interviews were conducted with providers and patients.

**Results:**

There were 2398 episodes of HTC during 18 021 clinic attendances (13.3%) between January and April 2011. The proportion of clinic attendees undergoing HTC was lowest in non-pregnant women (6.3%) and men (8.5%), compared with pregnant women (47.2%). Men had more advanced HIV infection than women (79.7% WHO stage 3 or 4 *vs.* 56.4%). Problems with WHO staging and access to CD4 counts affected ART eligibility assessments; only 48% completed ART eligibility assessment, and 54% of those reporting WHO stage 3/4 illnesses were not referred to start ART promptly. On multivariate analysis, HIV-positive pregnant women were significantly less likely to be referred directly for ART initiation (adjusted OR: 0.29, 95% CI: 0.13–0.63).

**Conclusions:**

These data show that provider-initiated testing and counselling (PITC) has not yet been fully implemented at primary care clinics. Suboptimal ART eligibility assessments and referral (reflecting the difficulties of WHO staging in primary care) mean that simplified eligibility assessment tools are required to reduce unnecessary delay and attrition in the pre-ART period. Simplified initiation criteria for pregnant women, as being introduced in Malawi, should improve linkage to ART.

## Introduction

By 2010, over 5 million HIV-infected individuals were receiving antiretroviral therapy (ART) in low and middle income countries (UNAIDS 2010), with rapid scale-up achieved in a number of sub-Saharan African countries_ENREF_2 (Vitoria *et al.* 2009). In the face of resource and infrastructural constraints, Malawi has demonstrated great success in scaling-up the national ART programmes using a decentralized model and with task-shifting of treatment delivery from physicians to other cadres of health providers (Lowrance *et al.* 2008). By December 2010, over 300 000 individuals had initiated treatment (Ministry of Health of Malawi 2010).

Despite these achievements, coverage of ART remains suboptimal both in Malawi and elsewhere. UNAIDS estimates that in sub-Saharan Africa, only 37% of adults in immediate need of ART (defined as having a CD4 count of <350 cells/μl) currently receive treatment (UNAIDS 2010). In Malawi, this figure is 48% (World Health Organization 2010). In addition, most patients do not receive ART until they have advanced immunosuppression (Bassett *et al.* 2010), increasing the risk of early ART mortality (Lawn *et al.* 2008), treatment complications, opportunistic infections and transmission of HIV to partners (Granich *et al.* 2009).

Provider-initiated HIV testing and counselling (PITC) is the main entry point to comprehensive HIV care (including ART) for individuals attending health facilities (World Health Organization 2007a). Compared with client-initiated HIV voluntary testing and counselling (VCT), PITC has been shown to be highly cost-effective (Walensky *et al.* 2011). Malawian National HIV care guidelines recommend that all individuals attending health facilities should be offered PITC (Ministry of Health 2008), but scale-up of this approach has been held back by human resources and infrastructure constraints.

One of the critical barriers to ART scale-up in public health programmes has been the low proportion of newly diagnosed HIV-positive individuals who successfully initiate ART or are retained in pre-ART care if they are not yet eligible (Tayler-Smith *et al.* 2010). Current estimates are that only one-third of newly diagnosed HIV-positive adults are retained in care and go on to initiate ART (Rosen & Fox 2011).

This study investigated HIV testing, ART eligibility assessment and subsequent referral for ART at two large primary health care clinics offering PITC and comprehensive HIV care in Blantyre, Malawi. The objectives were to describe the proportion of primary clinic attendees receiving HIV testing and counselling (HTC); to describe referral patterns following HIV diagnosis and investigate patient and provider factors associated with decisions to refer; and to identify current limitations in the health system that contribute to delayed initiation of ART.

## Methods

### Study Design

A prospective cohort study of adults attending primary health care clinics and undergoing HTC in Blantyre, Malawi was undertaken. Baseline data from clinic attendance records between January and April 2011 are presented. Cohort follow-up is ongoing.

### Study site

Two primary health care centres (Ndirande Health Centre and Chilomoni Health Centre) with a combined catchment population of about 168 000 adults were selected as study sites in the high-density residential areas of Blantyre. These are the major primary care providers of ART treatment, with 3637 and 2083 patients, respectively, receiving ART at the start of the study (Ministry of Health of Malawi 2010). Adult HIV prevalence in urban north Blantyre was approximately 18.5% (Choko *et al.* 2011).

### ART care pathway in Malawi

**Figure 1:**
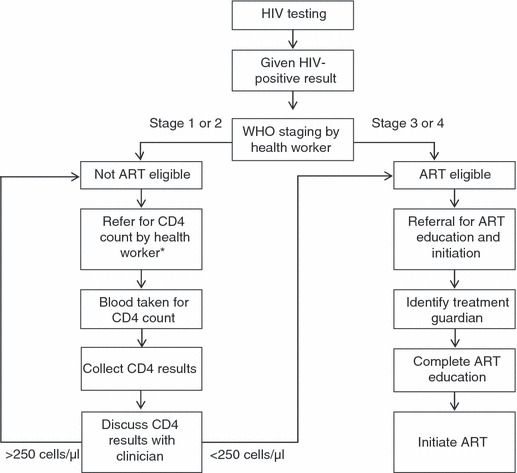
Antiretroviral therapy (ART) care pathway in Malawian National Programme. *After first occasion, WHO staging and CD4 are repeated 6-monthly for those not ART eligible.

Malawian national HIV care guidelines recommend that, following confirmation of HIV infection, patients undergo clinical staging based on the World Health Organization’s Clinical Staging System for HIV/AIDS (World Health Organization 2005, 2007b). Those in WHO stage 3 or 4 or with a CD4 count of <250 cells/μl are ‘ART eligible’ and should be referred directly for treatment education and ART initiation –Figure 1. Individuals in WHO stage 1 or 2 should be referred for venipuncture for CD4 count (in this population, at the nearby tertiary Queen Elizabeth Central Hospital). Patients then collect their CD4 count result and bring them to the primary health care centre for review. Those with a CD4 count of <250 cells/μl are ‘ART eligible’ and referred for treatment education and ART initiation at the same primary care clinic; those with a CD4 count of ≥250 cells/μl are ‘not ART eligible’ and should be ‘referred to the general health services for management and advice, for example, initiation of cotrimoxazole preventative therapy’ (Ministry of Health 2008).

At the time of the study, ART eligibility criteria for pregnant women were the same as for non-pregnant adults. Pregnant women not meeting these criteria were referred for prevention of mother to child therapy (PMTCT).

### Study participants and case definitions

Data on all clinic attendances and HIV testing events were extracted by research assistants from clinic registers. At each of the two clinics, newly diagnosed HIV-positive adults (≥16 years) were asked to participate in the study, with informed consent. Participants were interviewed immediately after completion of all HIV testing procedures and WHO staging (if completed). As result of the large number of clinic attendees and limited availability of private rooms, the maximum number of participants recruited per day was limited to six per clinic.

Participants underwent a baseline interview including questions on demographic and socioeconomic variables, previous HIV tests, knowledge and awareness of HIV and ART treatment, and their understanding of instructions given to them by the clinic staff regarding follow-up and referral appointments. Participants were then independently staged by study research assistants using the WHO clinical staging criteria (World Health Organization 2005).

Participants referred to treatment education prior to initiation of ART were classified as ‘referred directly for ART’. Participants referred for CD4 count measurement were classified as ‘referred for further ART assessment’. The participant’s understanding of instructions was confirmed by inspection of patient-carried health records and HIV testing registers. Where research staff identified poor understanding or misinterpretation, patients were referred back to clinical staff for clarification.

### HIV testing procedures

Routine HIV testing algorithms followed national guidelines (serial testing using Determine 1/2, Abbott, Tokyo, Japan, and, if positive, Uni-Gold HIV, Trinity Biotech, Dublin, Ireland, with SD Bioline 1/2, Standard Diagnostics, Korea if the first two were discordant).

### Statistical methods

Participant demographic and clinical characteristics were expressed as proportions, medians or means as appropriate and compared using chi square tests and Wilcoxon rank sum tests. Self-reported general health was recorded on a 4-item Likert-type scale (excellent, good, fair or poor). Referral for onward care following positive HIV diagnosis was expressed as a dichotomous variable (referral for ‘ART’ or ‘referral for further assessment’) and logistic regression models constructed to examine associations between participant characteristics and referral outcomes. Factors associated with incorrect referral (as assessed against study-performed WHO clinical staging) were examined using univariate logistic regression. Statistical analysis was undertaken using STATA v11.2 (Statacorp, College Station, TX, USA).

### Qualitative in-depth interviews

Study participants were purposively sampled to include a mix of men, pregnant and non-pregnant women and participants recruited from both study clinics. Health workers at the two clinics were purposively sampled to include those whose roles predominantly involved offering and performing HTC (counsellors) and those who predominantly undertook ART eligibility assessments and made referrals (clinicians), although there was some overlap in roles.

A trained qualitative research fieldworker undertook in-depth interviews based on semi-structured interview guide with participants and health workers. Interviews were audio recorded in Chichewa, translated into the English language and transcribed. A sample of 5% of transcripts was back-translated into Chichewa to check consistency. Coding of data was performed using NVIVO 9 (QSR, Melbourne, Australia). Analysis of themes used a framework approach.

### Ethical approval

Ethical approval was received from the Research Ethics committees of Liverpool School of Tropical Medicine and the College of Medicine of Malawi. Participants recruited to the study give informed written (or witnessed thumbprint) consent. Permission was obtained from the Blantyre District Health Officer and Nurses in Charge of both clinics for anonymized data collection from clinic registers.

## Results

### Clinic attendances during study period

A total of 18 021 adults (16 years or older) attended the two study clinics between January and April 2011 (Table 1). Clinic attendance was much more common by women [12 424/18 021 (68.9%)] than men [5597/18 021 (31.1%)]. This pattern remained after excluding attendances made by pregnant women [non-pregnant women-visits: 9625/15 222 (63.2%), male-visits: 5597/15 222 (36.8%)]. Ndirande Health Centre (located next to a busy urban market area) was busier than Chilomoni Health (located in a residential area).

**Table 1:** Clinic attendances during study period

	Chilomoni Health Centre 6348				Ndirande Health Centre 11 673			
		
	Women 4357		Men 1991		Women 8067		Men 3606	
General outpatients department	2967	68.1%	1718	86.3%	6028	74.7%	3364	93.3%
TB clinic	13	0.3%	15	0.8%	13	0.2%	26	0.7%
Antenatal clinic	460	10.6%	n/a	n/a	1019	12.6%	n/a	n/a
HIV testing and counselling clinic	917	21.1%	258	13.0%	1007	12.5%	216	6.0%

### HIV testing and counselling during clinic attendances

**Figure 2:**
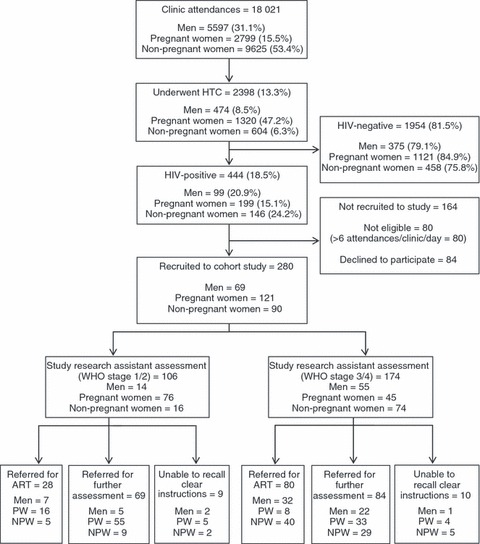
Clinic attendances, HIV test uptake and patterns of referral in participants recruited to cohort study. PW, pregnant women; NPW, non-pregnant women.

HIV testing and counselling was completed on 2398 of 18 021 (13.3%) clinic attendances (Figure 2). The highest rate of HTC was seen among pregnant women (1320/2799 attendances, 47.2%), but was substantially lower among men (474/5597 attendances, 8.5%) and non-pregnant women (604/9625 attendances, 6.3%). Individuals attending Chilomoni were significantly more likely to receive HTC than at Ndirande (18.5%*vs.*10.5%, *P* < 0.001 –Figure 2). A higher proportion of male attendees at Chilomoni underwent HTC compared with Ndirande (13.0%*vs.* 6.0%. *P* < 0.001).

### HIV testing outcomes

A total of 444 (18.5%) of 2398 attendees tested were HIV-positive. HIV prevalence was 19.2% (208/1175) in Ndirande compared with 17.7% (235/1223) in Chilomoni (*P* = 0.340). HIV prevalence was lowest in pregnant women (15.1%), followed by men (20.9%) and non-pregnant women (24.2%), *P* < 0.001: this may reflect higher rates of testing among pregnant attendees. HIV prevalence was significantly higher in men tested at Ndirande (55/216 [25.5%]) than at Chilomoni [44/258 (17.0%), *P* = 0.025].

### Baseline characteristics of participant recruited to cohort study

A total of 280 participants (76.9% of those eligible) were recruited to the study (Figure 2). Of the 164 individuals who were not recruited, 80 were not eligible, because six participants had already been recruited from the clinic on that day, and 84 patients did not consent to participation. Characteristics of participants closely reflected HIV-positive attendees. Forty-three per cent of participants were pregnant women, 25% were men and 32% were non-pregnant women; similar to the total population of clinic attendees testing HIV-positive.

Baseline characteristics of the 280 cohort study participants are shown in Table 2. There were high levels of illiteracy (18.9%) and high reported household food insecurity (20.7%). 85.5% men were in formal employment, but this was much lower in women (30.3%). Just over half (53.9%) of study participants had had a previous HIV test and 6 (2.1%) were currently being treated for TB. Men (71.0%) were significantly more likely to have undergone HTC for the first time than women (48.3%, *P* = 0.001). They also had worse self-reported general health: 66.7% self-reported their general health to be ‘fair’ or ‘poor’ compared with 36.0% of women (*P* < 0.001). On study assessed WHO staging, men had more advanced illness than women, with 79.7% of men in Stage 3 or 4, compared with 56.4% of women (*P* = 0.001).

**Table 2:** Baseline characteristic of HIV-positive clinic attendees recruited to cohort study

	Women (*n* = 211)		Men (*n* = 69)		*P*-value
Pregnant (ANC clinic)	121	57.3%	n/a	n/a	–
Age (years), median (IQR)	27	23–31	33	29–39	0.001
BMI (kg/m^2^) median (IQR)	23	20–24	21	19–22	0.001
Literacy					
Illiterate	48	22.7%	5	7.2%	0.004
Currently in formal employment					
Yes	64	30.3%	59	85.5%	<0.001
Self-reported general health					
Excellent	61	28.9%	8	11.6%	<0.001
Good	74	35.1%	15	21.7%	
Fair	61	28.9%	29	42.0%	
Poor	15	7.1%	17	24.6%	
WHO stage†					
Stage 1 or 2	92	43.6%	14	20.3%	0.001
Stage 3 or 4	119	56.4%	55	79.7%	
Reports household food insecurity‡					
Yes	43	20.4%	15	21.7%	0.809
First time tested for HIV					
Yes	109	51.7%	20	29.0%	0.001
Perceived difficulty in attending primary health care clinic					
Not difficult	23	11.1%	13	18.8%	0.096
Difficult	185	88.9%	56	81.2%	
How many family or household members do you know to be HIV-positive?					
None	120	56.9%	32	46.4%	0.129
One or more	91	43.1%	37	53.6%	
How many family or household members do you know who have ever taken ART?					
None	148	70.1%	44	63.8%	0.322
One or more	63	29.9%	25	36.2%	
How many family or household members do you know to have died from HIV/AIDS?					
None	153	72.5%	48	69.6%	0.637
One or more	58	27.5%	21	30.4%	
If I was told I needed to take ART, I would be happy to take it					
Agree	199	98.0%	68	100.0%	0.507
Disagree	2	1.0%	0	0.0%	

BMI, body mass index.

† WHO staging assessment as performed by study research assistant.

‡ Defined as skipping a meal in the last 2-weeks to ensure food.

### Awareness and readiness for ART

Participants had high awareness of HIV/AIDS, with nearly half (128/280, 45.7%) knowing at least one family or household member who had been diagnosed HIV-positive. Similarly, 31.4% (88/280) knew of at least one family member who had taken ART, and 28.2% (79/280) knew at least one family or household member who had died of HIV/AIDS. Readiness to take ART was high, with 98.5% (267/271) agreeing that they would take ART if referred by a health worker, and 87.1% (243/279) agreeing that they felt ready to start taking ART.

### Referral for ART and HIV care

Following confirmed HIV diagnosis, 108 participants (38.6%) were directly referred for ART by clinic staff, 153 (54.6%) were referred for further ART eligibility assessment (CD4 count) and 19 (6.8%) reported having received no clear instructions. Only 134 (48.2%) had undergone WHO clinical staging at the time of their HIV test, with the remainder asked to return by clinic staff for staging when clinicians were available.

Of the 174 participants in WHO stage 3 or 4, 80 (46.0%) were referred directly for ART by clinic staff with the remaining 54.0% referred instead for CD4 count measurement. Three quarters (78/106, 73.6%) of participants in WHO stage 1 or 2 were correctly referred for CD4 count measurement. On univariate logistic regression, participants diagnosed at Ndirande were less likely to be incorrectly referred than those at Chilomoni [odds ratio (OR): 0.60, 95% confidence interval (CI): 0.37–0.97]. However, the gender, age and pregnancy status of participants were not associated with incorrect referral (Table 3).

**Table 3:** Univariate associations with incorrect referral following HIV-positive diagnosis

	Proportion incorrectly referred (%)	Odds Ratio (95% CI)	*P*-value
Male sex	30/69 (43.5)	0.99 (0.58–1.72)	0.986
Pregnancy	53/121 (43.0)	1.02 (0.63–1.64)	0.946
Age (continuous)	–	1.00 (0.97–1.03)	0.956
BMI (kg/m^2^– continuous)	–	0.97 (0.91–1.05)	0.553
WHO stage			
Stage 1 or 2	28/106 (26.4)	1	
Stage 3 or 4	94/174 (54.0)	3.27 (1.94–5.53)	<0.001
Self-reported general health			
Excellent	24/69 (34.8)	1	0.185
Good	40/89 (44.9)	1.96 (1.03–3.74)	
Fair	46/90 (51.1)	1.53 (0.80–2.93)	
Poor	12/32 (37.5)	1.12 (0.47–2.69)	
Ndirande Health Centre	48/130 (36.9)	0.60 (0.37–0.97)	0.036
In formal employment	54/123 (43.9)	0.89 (0.38–2.09)	0.921
Illiterate	23/53 (43.4)	0.99 (0.54–1.81)	0.977
Household food insecurity†	23/58 (39.7)	0.82 (0.45–1.47)	0.498
First time tested for HIV	64/151 (42.4)	0.90 (0.56–1.45)	0.665
Patient perceived difficulty in attending clinic	20/36 (55.6)	0.36 (0.29–1.17)	0.125

BMI, body mass index.

† Defined as skipping a meal in the last 2-weeks to ensure food for children.

### Factors associated with direct referral for ART

On univariate analysis (Table 4), factors significantly associated with direct referral for ART included male sex (OR 2.6, 95% CI: 1.53–4.67), worse self-reported general health (*P*_trend_ < 0.001) and being in WHO Stage 3 or 4 (OR: 2.37, 95% CI: 1.40–4.01). Pregnant women were significantly less likely to be directly referred for ART (OR 0.22, 95% CI: 0.13–0.38).

**Table 4:** Univariate and multivariate associations with direct referral for ART

	Odds Ratio (95% CI)	Adj. Odds Ratio (95% CI)	*P*-value
Male sex	2.68 (1.53–4.67)	0.87 (0.43–1.75)	0.697
Pregnant	0.22 (0.13–0.38)	0.29 (0.13–0.63)	0.002
Age (continuous)	1.06 (1.03–1.10)	1.03 (1.00–1.07)	0.042
BMI (kg/m^2^– continuous)	0.85 (0.79–0.92)	0.95 (0.86–1.04)	0.057
Illiterate	1.28 (0.77–2.04)		
In formal employment	1.91 (1.17–3.11)†		
WHO stage‡			
Stage 1 or 2	1	1	0.997
Stage 3 or 4	2.37 (1.40–4.01)	1.00 (0.50–2.01)	
Self-reported general health			
Excellent	1	1	0.036
Good	1.90 (0.91–3.96)	2.43 (0.98–5.98)	
Fair	3.44 (1.68–7.05)	2.62 (1.15–5.93)	
Poor	10.04 (3.81–26.45)	4.55 (1.41–14.60)	
Clinic where HTC performed			
Chilomoni Health Centre	1	1	0.001
Ndirande Health Centre	1.61 (0.99–2.62)	2.82 (1.54–5.17)	
First time tested for HIV			
Yes	1.26 (0.77–2.04)		
Household food insecurity§			
Yes	1.06 (0.59–1.91)		
Patient perceived difficulty in attending clinic			
Difficult	0.77 (0.38–1.56)		

BMI, body mass index; HTC, HIV testing and counselling

† Not carried forward for multivariate analysis due to collinearity.

‡ WHO staging assessment as performed by study research assistants.

§ Defined as skipping a meal in the last 2-weeks to ensure food for children.

On multivariate adjustment, self-reported ‘poor’ (adjusted OR: 4.21, 95% CI: 1.35–13.07) and ‘fair’ (adjusted OR: 2.62, 95% CI: 1.15–5.93) general health remained significantly associated with direct referral for ART, whereas pregnancy (adjusted OR: 0.38, 95% CI: 0.18–0.78) was associated with referral for further ART assessment. WHO stage was not associated with direct referral for ART (adjusted OR: 0.91, 95% CI: 0.46–1.79), nor was male sex (adjusted OR: 0.97, 95% CI: 0.50–1.90). Attendees at Ndirande were more likely to be referred directly for ART initiation compared with those at Chilomoni (adjusted OR: 2.82, 95% CI: 1.54–5.17).

### Qualitative in-depth interviews

In total, 30 in-depth interviews were undertaken with study participants and 10 with healthcare providers. Qualitative analysis of clinic HIV testing practices showed that, rather than being offered HIV testing by providers when attending the clinic, non-pregnant participants tended to be diagnosed after a prolonged period of declining ill health that promoted self-presentation and request for HIV testing.

So I was examining myself and could see that my body is not alright. That is what made me think that aah its better to go where I hear that they do some tests – maybe I have a disease – That is why I mustered up boldness to go for testing.

Male participant, Chilomoni Health Centre.

Provider-initiated HIV testing and counselling seemed not to be fully implemented within the primary health care system, and instead, HIV testing operated on a more traditional VCT model, albeit situated in a health facility. In contrast, when attending for antenatal care, pregnant women understood that they would be tested for HIV. Acceptance of this approach seemed to be high.

When I came for antenatal clinic I was told that ‘Here we test for HIV, we test any person who is pregnant.

Female participant, pregnant, Ndirande Health Centre.

What they want is just to ask you ‘Are you ready?’ [to test] and we answer: Yes!

Female participant, pregnant, Ndirande Health Centre.

After diagnosis of HIV, participants described switching to a ‘dependant role’. Most had heard of ART, were aware of the potential benefits and wanted to start treatment rapidly. However, their care-seeking became dependent upon interactions with health workers (who were often rushed and provided little information), their families (from whom they had to borrow money to travel for CD4 count measurement and had to recruit as their ‘treatment guardian’) and the health system (long queues at health facilities, unreliable lab equipment and clinicians being intermittently available). As a result, many felt frustrated with the difficulties of getting CD4 measurements and results and some dropped-out of ART assessment.

So it took me time the way [Central Hospital] is – there are a lot of people. I went there in the morning, I happened to come back in the evening […]. When I went there in February my file got lost.’

Female participant, pregnant, Ndirande Health Centre.

Interviews with health workers predominantly focused on their experience of undertaking ART eligibility assessment and decision-making. They were candid about the difficulties in undertaking WHO staging, and acknowledged that time constraints forced them to refer patients for CD4 count measurement if unable to complete WHO staging during the time available for busy clinics.

Sometimes we have more than 80 clients, so out of that number, to do WHO staging for everybody, it seems as if we are delaying others. So, we just tell them in a short-cut way and refer them for CD4 count to ensure that everybody should feel that they have been helped in the right time.

Nurse, female, antenatal clinic, Ndirande Health Centre.

Despite these frustrations, health workers felt proud when they saw clients regain health following ART initiation.

## Discussion

The main findings from this study were the low proportion of clinic attendees offered PITC and the impact of health system constraints in ART eligibility assessment and referral for HIV care. Critical bottlenecks presenting major constraints to timely completion of ART eligibility assessment were the lack of CD4 count machines on-site, and the complexity and time taken to do the current clinical staging (WHO staging). Taken together, these findings show that there are significant limitations in the current provision of PITC and access to comprehensive HIV care in the primary health care system that could impair efforts to ensure universal knowledge of HIV status and access to ART.

Provider-initiated HIV testing and counselling [which is recommended by WHO for countries with generalized HIV epidemics (World Health Organization 2007a)] differs from client-initiated VCT, in that providers are responsible for recommending HIV testing to all health facility attendees, ‘even if they do not have obvious HIV-related symptoms or signs’ (World Health Organization 2007a). In the VCT approach, individuals who recognize themselves to be unwell request testing. Compared with VCT, PITC has been shown to be quicker, to result in a substantially higher proportion of clinic attendees agreeing to test (Braitstein *et al.* 2008; Harries *et al.* 2010; Topp *et al.* 2011)_ENREF_18 and to be acceptable for clients and providers (Obermeyer & Osborn 2007).

This study found that only 13% of all adult clinic attendees underwent HTC, with particularly low proportions seen in men and non-pregnant women. This is substantially lower than would have been expected if PITC had been fully implemented in the health system (Topp *et al.* 2011). To increase uptake of HTC among clinic attendees, guidelines recommending PITC should be fully put into practice, with additional commitments made to appropriately resourcing this approach including increasing availability of HTC trained health workers and minimizing HIV test kit stock-outs. To streamline PITC, opt-out blood sampling could be undertaken at clinic registration, with post-test counselling provided during the subsequent clinical consultation.

Of particular concern was the high proportion of men who were diagnosed HIV-positive at an advanced stage of disease (80% in WHO stage 3 or 4). Men were more likely to be referred directly for ART initiation than women. This suggests that the advanced degree of immunosuppression at which men initiate ART (Braitstein *et al.* 2008), and their higher early ART mortality (Lawn *et al.* 2008), are resultant upon late diagnosis of HIV and possible failure of linkage between testing and treatment (Bassett *et al.* 2010), rather than health worker referral patterns. Scale-up of strategies to encourage men to test more frequently and at an earlier stage of illness [including full implementation of PITC, ideally supported by workplace (Corbett *et al.* 2006) and home-based testing programmes (Ganguli *et al.* 2009)] are urgently required.

Provider-initiated HIV testing and counselling was more fully implemented in the antenatal clinic, with a higher proportion of pregnant attendees undergoing HTC (47%). In Malawi in 2010, 69% of ANC clinic attendees had their HIV status ascertained during the course of their pregnancy (Ministry of Health of Malawi 2010). Most pregnant women make more than one antenatal visit during the course of their pregnancy, and so, our figure will underestimate uptake of HTC.

Pregnancy was found to be significantly associated with 71% lower odds of direct referral for ART after adjusting for other confounders that may have associated with pregnant women’s propensity to be tested earlier in their disease course through antenatal care. We speculate based on the qualitative interviews with healthcare providers that pregnant women may have been referred to PMTCT rather than for ART initiation due to difficulties in completing WHO clinical staging. Universal combination ART for pregnant women, as is being implemented in Malawi (PMTCT B+) (Schouten *et al.* 2011), will substantially simplify the process of ART eligibility assessment in pregnancy.

ART eligibility assessment and referral following HIV diagnosis were found to be suboptimal, despite participant’s familiarity with HIV and ART and readiness to initiate treatment. Forty-four per cent of participants were incorrectly referred with 54% of those in WHO stage 3 or 4 referred for CD4 count measurement instead of being referred directly to start ART. We also noted differences in ART referral outcomes between clinics, suggesting that health worker and health system factors will be important to address in improving linkage to ART. Critical stumbling blocks identified were the difficulties and cost to clients of obtaining CD4 count measurements and, for providers, the complexity and length of WHO clinical staging.

The World Health Organization’s Clinical Staging System for HIV/AIDS was revised in 2005 and has a sensitivity of 51–52% and specificity of 68–88% in predicting CD4 count of <200 cells/mm^3^ (Kagaayi *et al.* 2007; Jaffar *et al.* 2008). However, it was initially designed as a specific and low-cost means of diagnosing patients with AIDS prior to the widespread availability of point of care diagnostic HIV tests (Gilks 1990). Our quantitative and qualitative data show that a simplified clinical staging approach to ART eligibility assessment that can be utilized in primary care may be required to ensure that patients are managed appropriately. At the time of this study, the CD4 threshold for initiation in Malawi was <250 cells/μl. Since completion of the study, the threshold has been raised to <350 cells/μl; however, the difficulties observed in completing CD4 count measurement at primary care level will be difficult to overcome without innovative technologies. The anticipated scale-up of technologies such as point of care CD4 count analysis systems may circumvent these problems, if widely implemented (Zachariah *et al.* 2011).

### Limitations

Despite the large numbers of clinic attendances examined, this study was conducted over a relatively short time period. Health system factors, such as staffing levels and availability of HIV test kits, at the time of the study, may have influenced findings. We did not take CD4 measurements at the time of HIV diagnosis, as the purpose was to describe the patterns of referral under programmatic conditions, and doing so would have been an intervention.

### Relevance of findings for improving linkage from HIV diagnosis to ART initiation

There is increasing evidence that expanded access to ART, and initiation at higher CD4 count thresholds will have large public health benefits (Granich *et al.* 2009). This has turned attention towards the high rates of attrition seen between HIV diagnosis and initiation of ART, with some studies suggesting that only between one-sixth and one-third of patients will go on to initiate ART (Bassett *et al.* 2010; Rosen & Fox 2011; Topp *et al.* 2011). Findings from this study go some way to explaining the underlying reasons for poor linkage in the immediate post-diagnosis period in Malawi and will need to be confirmed in other high HIV prevalence settings. Considerable care will be required to ensure that HIV testing and ART programmes are structured to facilitate rapid and uncomplicated access to treatment.

## Conclusions

In summary, we report on low levels of PITC (especially for men and non-pregnant women), suboptimal ART eligibility assessments and deficiencies in patterns of referral for ART among clinic attendees in primary care (with pregnant women particularly disadvantaged). Of particular concern was that assessment of ART eligibly using the WHO Clinical Staging System did not seem ideally suited to busy primary health care clinic clinics. These findings contribute to understanding of the poor rates of linkage and long delays between HIV diagnosis and ART initiation.
